# Comparative Analysis of Streptozotocin, Streptozotocin–Nicotinamide and Alloxan-Based Diabetes Models in Female Wistar Rats

**DOI:** 10.3390/mps9030072

**Published:** 2026-05-02

**Authors:** Sabrina-Gabriela Mîndruț, Cristina Pop, Sorin-Marian Mârza, Alexia-Teodora Hoța, Flaviu-Alexandru Tăbăran, Ibrahima Mamadou Sall, Ana Uifălean, Emilia-Laura Mogoșan, Oliviu Voștinaru, Cristina-Ionela Mogoșan

**Affiliations:** 1 Department of Pharmacology, Physiology and Physiopathology, Faculty of Pharmacy, “Iuliu Hațieganu” University of Medicine and Pharmacy, 400347 Cluj-Napoca, Romania; sabrina.gabr.mindrut@elearn.umfcluj.ro (S.-G.M.); cmogosan@umfcluj.ro (C.-I.M.); 2 Clinics Department, Faculty of Veterinary Medicine, University of Agricultural Sciences and Veterinary Medicine, 400372 Cluj-Napoca, Romania; sorin.marza@usamvcluj.ro; 3 Department of Pathological Anatomy, Faculty of Veterinary Medicine, University of Agricultural Sciences and Veterinary Medicine, 400372 Cluj-Napoca, Romania; alexia-teodora.hota@usamvcluj.ro (A.-T.H.); ibrahima.sall@student.usamvcluj.ro (I.M.S.); 4 Department of Morpho-Functional Sciences, Discipline of Pathophysiology, “Iuliu Hațieganu” University of Medicine and Pharmacy, 400012 Cluj-Napoca, Romania

**Keywords:** streptozotocin, alloxan, nicotinamide, experimental diabetes, female Wistar rats, oxidative stress, oral glucose tolerance test, hepatic, pancreatic and renal histopathology

## Abstract

Experimental diabetes models induced by streptozotocin (STZ) and alloxan are widely used in preclinical research; however, direct standardized comparisons in female rodents remain limited. The present study evaluated multiple chemical induction protocols in female Wistar rats, including STZ (40 and 65 mg/kg), STZ at the same doses combined with nicotinamide (110 mg/kg), and alloxan (130 mg/kg). Glycemic progression, oral glucose tolerance test, body weight evolution, oxidative stress markers, and multi-organ histopathology were assessed over a 14-day period. High-dose STZ (65 mg/kg) and alloxan produced rapid, sustained hyperglycemia (*p* < 0.0001), significant body weight reduction, increased lipid peroxidation (elevated MDA), nitric oxide overproduction, thiol depletion, and pronounced pancreatic and renal structural damage. In contrast, STZ–nicotinamide protocols generated moderate but stable hyperglycemia with partial preservation of islet architecture, attenuated oxidative imbalance, and improved systemic tolerability. Oral glucose tolerance test confirmed impaired glucose handling in the STZ–nicotinamide group, consistent with a type 2 diabetes-like phenotype rather than complete insulin deficiency. These results demonstrate that induction strategy critically determines metabolic stability, oxidative stress burden, and tissue remodeling patterns, supporting model selection according to specific experimental objectives.

## 1. Introduction

Diabetes mellitus (DM) is a major global public health challenge with continuously increasing prevalence and major clinical, social, and economic consequences. According to the International Diabetes Federation (IDF) Diabetes Atlas, 11th edition, approximately 589 million adults aged 20–79 years were living with diabetes in 2024, and this number is projected to rise to 853 million by 2050. The global burden is particularly concerning because a substantial proportion of cases remain undiagnosed and because diabetes is associated with long-term microvascular and macrovascular complications that significantly reduce quality of life and increase mortality [1].

According to the American Diabetes Association (ADA), diabetes is classified into several clinical categories, including Type 1 diabetes mellitus (T1DM), Type 2 diabetes mellitus (T2DM), gestational diabetes mellitus, and other specific types of diabetes. It is a chronic metabolic disorder characterized by hyperglycemia resulting from defects in insulin secretion, insulin action, or both. Persistent hyperglycemia contributes to the development of cardiovascular disease, nephropathy, neuropathy, retinopathy, and other systemic complications [2].

Experimental models are essential for understanding diabetes pathophysiology and for evaluating novel therapeutic strategies. Among available approaches, chemically induced diabetes models remain widely used because they are relatively simple, reproducible, and cost-effective. Streptozotocin (STZ) and alloxan are the most frequently used diabetogenic agents, although they differ in their mechanisms of beta-cell injury and in the severity and stability of the induced metabolic phenotype [3].

In the present study, both severe insulin-deficient models and moderate diabetes-like models were evaluated. STZ alone, especially at the higher dose, and alloxan were used as severe beta-cell toxic models resembling type 1 diabetes-like conditions, whereas STZ combined with nicotinamide was used as a moderate model with partial beta-cell preservation, more closely resembling type 2 diabetes-like metabolic dysfunction. This distinction is important for selecting an experimental protocol according to the specific scientific objective.

Despite the extensive use of rodent models in diabetes research, females remain underrepresented in preclinical studies, as male animals are often preferred to avoid hormonal variability related to the estrous cycle [4]. However, growing evidence indicates that sex significantly influences glucose metabolism, insulin sensitivity, oxidative stress, inflammatory responses, and the severity of diabetic complications [5]. Experimental and translational studies suggest that estrogen may exert partial protective effects on beta-cell injury, oxidative stress, and tissue remodeling, which may alter both susceptibility to diabetes induction and disease progression [6,7]. Therefore, the characterization of diabetes models in female animals is important not despite hormonal variability, but precisely because sex-specific biological responses may influence experimental outcomes and translational relevance [8].

Although STZ-, STZ–nicotinamide-, and alloxan-induced diabetes models are widely described in the literature, direct head-to-head comparisons performed under standardized experimental conditions remain limited, particularly in female rodents. The present study was designed to address this gap by comparatively evaluating commonly used chemical induction protocols in female Wistar rats using fasting glycemia, oral glucose tolerance testing, body weight evolution, oxidative stress markers, and histopathological assessment [9].

Research into T1DM remains of major scientific relevance due to its autoimmune etiology and rapid progression toward absolute insulin deficiency. T1DM is characterized by immune-mediated destruction of pancreatic β-cells, leading to lifelong dependence on exogenous insulin and increased risk of acute metabolic complications. Although Type 2 T2DM accounts for approximately 90–95% of adult diabetes cases worldwide [10], T1DM represents a critical model for understanding β-cell failure, immune dysregulation, and early-onset metabolic instability. Moreover, mechanistic insights gained from T1DM research have contributed substantially to advances in immunomodulatory strategies and β-cell preservation approaches [11]. The systemic consequences of insulin deficiency in T1DM also provide a valuable framework for studying multi-organ diabetic complications, including cardiovascular, renal, and neurological alterations [12].

Streptozotocin (STZ) and alloxan are the most commonly employed diabetogenic agents, both preferentially targeting pancreatic β-cells through different mechanisms, leading to insulin deficiency and hyperglycemia. Streptozotocin induces β-cell death primarily through DNA alkylation following cellular uptake via the GLUT2 transporter, leading to activation of poly(ADP-ribose) polymerase (PARP), NAD^+^ depletion, and subsequent ATP exhaustion. In contrast, alloxan exerts its diabetogenic effect predominantly through redox cycling and rapid generation of reactive oxygen species, resulting in oxidative damage, intracellular calcium dysregulation, and selective β-cell necrosis. However, the severity and stability of the induced diabetic phenotype depend strongly on the induction protocol, including dose and other experimental conditions [13].

High-dose STZ or alloxan frequently produce aggressive models with marked weight loss and increased mortality, which may limit their suitability for studies requiring stable long-term follow-up. To obtain milder diabetic phenotypes, either lower doses of STZ were used or nicotinamide (NA) has been combined with STZ due to its partial protective effect on β-cells, resulting in more moderate hyperglycemia and improved tolerability [14,15].

Therefore, direct comparisons of commonly used induction protocols are important for selecting the most appropriate model for specific research objectives.

In this study, several chemical protocols for diabetes induction in female Wistar rats were compared, including different STZ doses, STZ–NA combinations, and alloxan administration, using fasting blood glucose monitoring, body weight assessment, and oral glucose tolerance testing.

Although streptozotocin-, streptozotocin–nicotinamide-, and alloxan-induced diabetes models are widely described in the literature, direct head-to-head comparisons under strictly standardized experimental conditions remain limited, particularly in female rodents. Most previous studies have either focused on a single induction protocol or have compared models using heterogeneous housing conditions, variable timelines, or distinct biochemical endpoints, making cross-study interpretation difficult.

The present study provides a controlled, side-by-side evaluation of commonly used chemical induction strategies in female Wistar rats, integrating fasting glycemia, oral glucose tolerance testing, oxidative stress profiling, and multi-organ histopathological assessment within the same experimental framework. By correlating metabolic severity with redox imbalance and tissue remodeling patterns, this work aims not only to compare induction efficacy but also to offer a practical decision-oriented framework to guide protocol selection according to research objectives. This integrative approach enhances reproducibility and supports more rational model selection in preclinical diabetes research.

From a pathophysiological perspective, the present work does not evaluate a single diabetes phenotype. Instead, it compares severe chemically induced insulin-deficient models (STZ65 and alloxan, and to a lesser extent STZ40) with moderate models characterized by partial beta-cell preservation (STZ40+NA and STZ65+NA). Accordingly, the study aims to distinguish protocols more appropriate for type 1 diabetes-like investigations from those more suitable for type 2 diabetes-like studies involving residual beta-cell function.

## 2. Materials and Methods

### 2.1. Chemicals and Reagents

Streptozotocin (STZ; 500 mg, S-0130, Sigma-Aldrich, St. Louis, MO, USA), nicotinamide (NA; 100 g, N-3376, Sigma-Aldrich, St. Louis, MO, USA), and alloxan monohydrate (ALO; Sigma-Aldrich, St. Louis, MO, USA) were used for diabetes induction. Sodium citrate buffer (0.5 M, pH 5.0; 250 mL, Thermo Fisher Scientific, Waltham, MA, USA), 10% glucose solution, distilled water, and sodium chloride solution (NaCl 0.9%) were also used throughout the experimental procedures. All reagents were of analytical grade and were prepared and handled according to the manufacturers’ instructions.

### 2.2. Experimental Animals

A total of 42 adult female Wistar albino rats (body weight 250–300 g) were used. Animals were obtained from the animal facility (Biobase—Center for Experimental Medicine and Practical Skills) of the “Iuliu Hațieganu” University of Medicine and Pharmacy, Cluj-Napoca, Romania. The experimental protocol was approved by the Institutional Animal Ethics Committee of the “Iuliu Hațieganu” University of Medicine and Pharmacy, Cluj-Napoca, Romania, and authorized by the National Sanitary Veterinary and Food Safety Authority (approval no. 456/25 July 2025).

Animals were housed 3–4 per cage, under standard laboratory conditions, with a 12 h light/dark cycle, ambient temperature maintained at 22–24 °C, and relative humidity of 55–60%. Standard pelleted chow and water were provided ad libitum throughout the study.

### 2.3. Experimental Design

#### 2.3.1. Experimental Groups and Induction of Diabetes

After a period of 7 days of acclimatization, diabetes mellitus (DM) was induced on the first experimental day following an overnight fast (12 h), with free access to water. Rats were randomly allocated into six experimental groups (*n* = 7 per group), using a random number generator to minimize allocation bias. Depending on group allocation, diabetes was induced using streptozotocin, streptozotocin combined with nicotinamide, or alloxan. The control group (CO) received intraperitoneal administration of 0.9% sodium chloride solution. STZ40 group received 40 mg/kg streptozotocin (STZ) intraperitoneally (i.p.), while STZ40+NA group received the same dose of STZ (40 mg/kg, i.p.) in combination with 110 mg/kg nicotinamide (NA). STZ65 group was treated with a higher dose of STZ (65 mg/kg, i.p.), whereas STZ65+NA received 65 mg/kg (i.p.) STZ combined with 110 mg/kg NA. ALO130 group received 130 mg/kg alloxan monohydrate (i.p.).

##### Streptozotocin-Only Diabetes Model

All animals were fasted overnight for 12 h before diabetes induction, with free access to water. In the STZ-only groups, rats received a single intraperitoneal injection of STZ at doses of 40 mg/kg or 65 mg/kg body weight.

STZ was freshly prepared immediately before administration by dissolution in cold sodium citrate buffer (0.5 M, pH 5.0), protected from light with aluminum foil, and kept on ice. Due to its chemical instability, STZ was administered within 10–15 min of preparation. To prevent early hypoglycemia, animals received a 10% glucose solution for 24 h after injection [16].

##### Streptozotocin–Nicotinamide Diabetes Model

For the STZ+NA models, rats were fasted for 12 h and weighed to calculate appropriate doses. Nicotinamide (110 mg/kg, i.p.) was administered first, followed 15 min later by STZ (40 mg/kg or 65 mg/kg, i.p.). STZ preparation and handling were performed as described above. Following induction, animals received 10% glucose solution for the first 24 h to minimize the risk of acute hypoglycemia [17].

##### Alloxan Diabetes

Rats in the ALO130 group were fasted for 12 h prior to induction. Diabetes was induced by a single intraperitoneal injection of alloxan monohydrate at a dose of 130 mg/kg body weight. After alloxan administration, animals received 10% glucose solution for 24 h to reduce the risk of severe hypoglycemia and were closely monitored throughout the experimental period [18].

#### 2.3.2. Monitoring of Glycemic Status and Body Weight

Following diabetes induction, animals were monitored for two weeks. For glycemic monitoring, rats were fasted for 12 h before each blood glucose determination, with free access to water. Capillary blood samples were collected from the tail vein using sterile lancets, and glucose concentrations were measured using a handheld glucometer (OneTouch Select Plus, LifeScan Inc., Milpitas, CA, USA), according to the manufacturer’s instructions.

Blood glucose was assessed at baseline (day 0, before diabetogenic agent administration), at 2 h post-induction to capture the early acute glycemic response, and subsequently on days 3, 7 and 14 after induction. Final fasting blood glucose was also recorded immediately prior to sacrifice.

Body weight was monitored as an additional indicator of metabolic status, systemic tolerability, and disease severity throughout the experimental period.

#### 2.3.3. Methodology of the Oral Glucose Tolerance Test (OGTT)

Before the end of the experiment, an oral glucose tolerance test (OGTT) was performed in the CO and STZ40+NA groups to evaluate glucose homeostasis. Rats were fasted overnight prior to the test, with free access to water. A 40% glucose solution was administered orally by gavage, and blood glucose levels were measured from tail vein capillary blood at 0 (baseline), 30, 60, 90, and 120 min after glucose administration. Glycemic responses were plotted as glucose concentration versus time and compared between the Control and STZ40+NA groups.

#### 2.3.4. Euthanasia and Sample Collection

At the end of the experimental period, animals were euthanized under deep anesthesia induced with ketamine (80–100 mg/kg) and xylazine (10 mg/kg), administered intraperitoneally, followed by cardiac puncture and exsanguination. Prior to euthanasia, final body weight and fasting blood glucose levels were recorded. Immediately after sacrifice, blood samples were collected via cardiac puncture under aseptic conditions. Samples were then centrifuged at 3000 rpm for 10 min at 4 °C, and the obtained serum and plasma were aliquoted and stored at −80 °C until biochemical analysis.

Tissue samples (pancreas, liver, and kidney) were rapidly excised, rinsed in cold saline, and processed according to their intended analysis. Tissue samples were either fixed in 10% buffered formalin, either stored at −80 °C until further processing.

#### 2.3.5. Oxidative Stress Markers

Oxidative stress was evaluated using complementary markers reflecting total oxidant burden, lipid peroxidation, non-enzymatic antioxidant reserve, and nitrosative stress.

In the present study, total oxidative status (TOS) was quantified using a ferrous ion oxidation–xylenol orange-based colorimetric assay, in which oxidant species present in plasma convert Fe^2+^ to Fe^3+^ under acidic conditions. The generated ferric ions form a colored complex with xylenol orange, proportional to the total oxidant burden and expressed as μM H_2_O_2_ equivalents/L [19].

This parameter provides an integrated estimate of the overall oxidant load present in the biological sample.

Serum malondialdehyde (MDA), a widely used surrogate surrogate marker of lipid peroxidation and membrane oxidative damage, was quantified using a modified thiobarbituric acid reactive substances (TBARS) assay. Briefly, serum samples were precipitated with trichloroacetic acid in the presence of EDTA and sodium dodecyl sulfate, while butylated hydroxytoluene was added to minimize ex vivo oxidation. Following centrifugation, the supernatant was reacted with thiobarbituric acid and heated at 95 °C to generate the MDA–TBA chromogenic adduct. Absorbance was measured at 532 nm, and concentrations were calculated from a 1,1,3,3-tetrahydroxypropane standard curve, expressed as nM/mL serum [20].

Total thiol groups (SH), reflecting non-enzymatic antioxidant reserves, were quantified using Ellman’s reagent (5,5′-dithiobis-(2-nitrobenzoic acid), DTNB). Thiol-containing compounds react with DTNB to produce a yellow-colored 5-thio-2-nitrobenzoic acid anion, measured spectrophotometrically at 412 nm. Results were expressed as μmol/L [21].

Total thiol groups reflect non-enzymatic antioxidant defenses, particularly reduced sulfhydryl-containing compounds.

Nitric oxide (NOx) levels were estimated indirectly by measuring total nitrate/nitrite concentrations using a colorimetric Griess reaction. Following enzymatic or chemical reduction of nitrate to nitrite, samples were reacted with Griess reagents to form a stable azo dye, quantified at 540 nm. NO concentrations were calculated using a sodium nitrite standard curve and expressed as μmol/L [22].

Nitric oxide-derived metabolites provide an indirect measure of nitrosative stress and inflammatory activation.

#### 2.3.6. Histological Analysis

Histological evaluation of pancreatic, hepatic, and renal tissues was performed using standard paraffin-embedding procedures. After fixation in 10% buffered formalin, tissues were dehydrated through graded ethanol series (70%, 80%, 90%, 96%, and 100%), cleared in xylene, and embedded in paraffin.

Paraffin blocks were sectioned at approximately 2 µm using a rotary microtome. Sections were mounted on glass slides and stained with hematoxylin and eosin (H&E) according to routine laboratory procedures. Histological evaluation was performed using an Olympus BX51 light microscope. Representative bright-field images were captured with an Olympus SP350 digital camera and processed using Olympus cellSens software (version 3.1) [23].

Lesions were assessed semi-quantitatively for each organ. The incidence of each histopathological change was recorded as the number of affected animals per group. Severity was expressed as the proportion of affected animals relative to the total number of animals in the respective group. Evaluated renal lesions included progressive nephropathy, inflammatory cell infiltrates, tubular degeneration or necrosis, casts, mineralization, and glomerular alterations. Hepatic assessment focused on inflammatory infiltrates, biliary hyperplasia, and mitotic activity. Pancreatic evaluation included islet cellularity, fibrosis, inflammatory infiltrates, degenerative changes, and exocrine alterations.

All evaluations were performed in a blinded manner to minimize observational bias.

#### 2.3.7. Statistical Analysis

Statistical analysis was performed using GraphPad Prism (GraphPad Software, San Diego, CA, USA). Data are presented as mean ± standard error of the mean (SEM). For comparisons among multiple experimental groups at a single time point, one-way analysis of variance (ANOVA) followed by Tukey’s post hoc test was used. For body weight analysis, two-way ANOVA was applied, with time and treatment group as factors, followed by an appropriate multiple-comparison post hoc test. A *p* value < 0.05 was considered statistically significant.

## 3. Results

### 3.1. Blood Glucose (Glycemia) Progression

**Figure 1:**
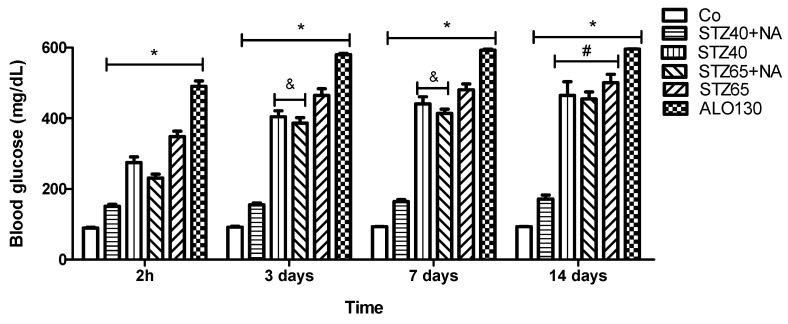
Fasting blood glucose levels (mg/dL) measured at baseline (day 0), 2 h after induction, and on days 3, 7 and 14 in the Co, STZ40, STZ40+NA, STZ65, STZ65+NA, and ALO130 groups (*n* = 7/group). * *p* ≤ 0.001 (Co vs. STZ40, STZ40+NA, STZ65, STZ65+NA, and ALO130 groups), ^&^ *p* ≥ 0.05 (STZ40 vs. STZ65+NA), ^#^ *p* ≥ 0.05 (STZ40 vs. STZ65+NA vs. STZ65).

Blood glucose levels were assessed at 2 h, 3 days, 7 days, and 14 days in six experimental groups. Data are expressed as mean ± SD. Statistical comparisons among groups at each time point were performed using one-way ANOVA followed by Tukey’s post hoc test (Figure 1).

At 2 h post-administration, a highly significant difference between treated groups and Co group was observed (F(5,32) = 670.6, *p* < 0.0001; R^2^ = 0.9905), indicating that treatment condition accounted for nearly all variability in glycemia. Yhis difference was maintained throughout the experiment. Mean glucose levels (mg/dL, mean ± SD) were 89.4 ± 5.59 in the CO, 150.71 ± 15.14 in STZ40+NA, 274.67 ± 39.37 in STZ40, 230.83 ± 26.48 in STZ65+NA, 348.10 ± 49.59 in STZ65, and 490.50 ± 30.65 in ALO130 animals. A clear dose-dependent effect was evident, with STZ65 producing more pronounced hyperglycemia than STZ40 (*p* ≤ 0.05). Nicotinamide co-administration attenuated the acute glycemic rise compared to STZ alone, whereas ALO130 animals exhibited the highest early glucose levels, suggesting rapid metabolic destabilization.

At 3 days, intergroup differences further intensified (F(5,30) = 873.0, *p* < 0.0001; R^2^ = 0.9932). Mean glycemia increased to 91.6 ± 5.73 mg/dL in CO, 155.4 ± 10.98 in STZ40+NA, 404.17 ± 41.33 in STZ40, 386.17 ± 38.29 in STZ65+NA, 464.73 ± 63.44 in STZ65, and 580.00 ± 7.83 in ALO130. Compared with the 2 h timepoint, glucose values rose markedly in all diabetic groups, indicating consolidation of β-cell dysfunction and progression toward established hyperglycemia. In the STZ65 group, higher glycemia compared to that obtained in the STZ40 suggests dose dependency. Although nicotinamide moderated glycemic elevation, for example in the STZ40 and STZ65+NA groups where glycemia levels were comparable (*p* ≥ 0.05), glycemia values in the NA treated groups remained substantially above control levels. The ALO130 group maintained the highest glucose concentrations, reflecting persistent and progressive metabolic dysregulation.

By day 7, maximal statistical separation was observed (F(5,28) = 1086, *p* < 0.0001; R^2^ = 0.9949), suggesting peak phenotypic stabilization. Mean glucose levels reached 93.0 ± 1.58 mg/dL in CO, 164.40 ± 11.67 in STZ40+NA, 440.80 ± 44.11 in STZ40, 413.50 ± 24.96 in STZ65+NA, 480.64 ± 55.25 in STZ65, and 592.75 ± 6.95 in ALO130. The progressive increase in F-statistics from 2 h to day 7 indicates amplification of intergroup divergence over time. Hyperglycemia became stable and sustained in STZ40, STZ65, and ALO130 animals, while nicotinamide continued to exert a moderating but incomplete protective effect.

At 14 days, significant intergroup differences persisted (F(5,32) = 495.4, *p* < 0.0001; R^2^ = 0.9872). Mean glucose levels were 93.0 ± 1.22 mg/dL in CO, 171.00 ± 31.70 in STZ40+NA, 464.60 ± 87.01 in STZ40, 454.50 ± 49.37 in STZ65+NA, 500.82 ± 78.95 in STZ65, and 596.00 ± 6.06 in ALO130. Although the F-value decreased compared with day 7, treatment effects remained robust, and hyperglycemia persisted in all diabetic groups. STZ65 and ALO130 animals displayed the most severe and sustained glycemic profiles, whereas STZ40 maintained a moderate-to-severe diabetic state. The convergence of STZ65+NA toward STZ40 levels at later stages suggests partial attenuation of high-dose STZ toxicity by nicotinamide, without full metabolic normalization.

Overall, the temporal evolution of F-statistics (670.6 → 873.0 → 1086 → 495.4) and consistently high R^2^ values (0.9872–0.9949) indicate that treatment condition was the dominant determinant of glycemic variability throughout the study. The data demonstrate rapid induction, progressive amplification, and long-term stabilization of hyperglycemia, with clear dose dependency, partial modulation by nicotinamide, and persistent severe metabolic disturbance in ALO130 animals. These findings confirm robust establishment and maintenance of the experimental diabetic phenotype across the 14-day observation period.

### 3.2. Results of the Oral Glucose Tolerance Test (OGTT)

The oral glucose tolerance test (OGTT) was performed to evaluate dynamic glucose handling and to differentiate between complete insulin deficiency and partial β-cell dysfunction. While fasting glycemia reflects basal metabolic disturbance, OGTT provides functional insight into residual insulin secretion capacity and peripheral glucose disposal.

**Figure 2:**
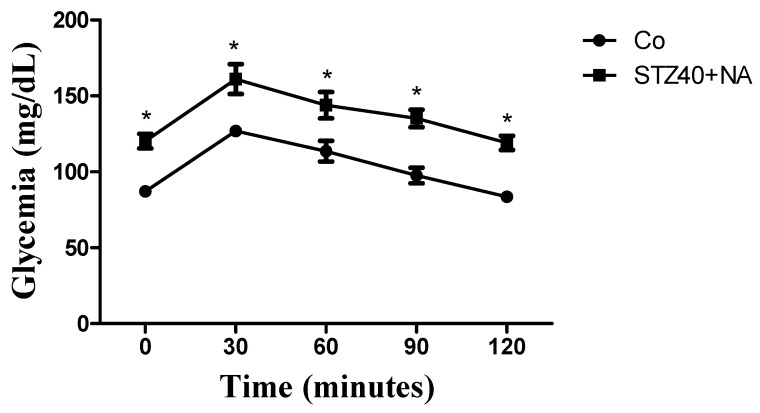
Oral glucose tolerance test (OGTT) performed in the CO and STZ40+NA groups after overnight fasting. * *p* ≤ 0.05.

In the present study, OGTT was conducted in CO and STZ40+NA groups. Blood glucose levels were measured before oral glucose administration and at 30, 60, 90, and 120 min after glucose loading. Data are expressed as mean ± SD (Figure 2).

The STZ+NA protocol was specifically selected for dynamic assessment because it is considered a moderate diabetes model characterized by partial β-cell preservation. Severe models (high-dose STZ and alloxan) were not subjected to OGTT due to the risk of excessive glycemic destabilization and limited physiological relevance in conditions of near-complete insulin deficiency.

Control animals exhibited a typical physiological response, characterized by a peak in glycemia at 30 min followed by gradual return toward baseline values by 120 min. In contrast, the STZ40+NA group demonstrated delayed glucose clearance and sustained post-load hyperglycemia, consistent with impaired glucose tolerance rather than absolute insulin absence.

These findings support the classification of the STZ+NA protocol as a moderate, type 2 diabetes-like phenotype, in which dynamic glucose dysregulation occurs in the presence of residual insulin activity. Therefore, OGTT served not merely as a confirmatory test of hyperglycemia but as a functional discriminator between moderate and severe induction strategies.

Because the primary objective of the OGTT in the present study was to functionally illustrate delayed glucose clearance in the STZ40+NA group compared with CO, interpretation focused on the temporal glycemic pattern rather than on area-under-the-curve (AUC) quantification. Future studies may incorporate AUC analysis to allow a more formal quantitative comparison of glucose excursion.

### 3.3. Body Weight Evolution

**Figure 3:**
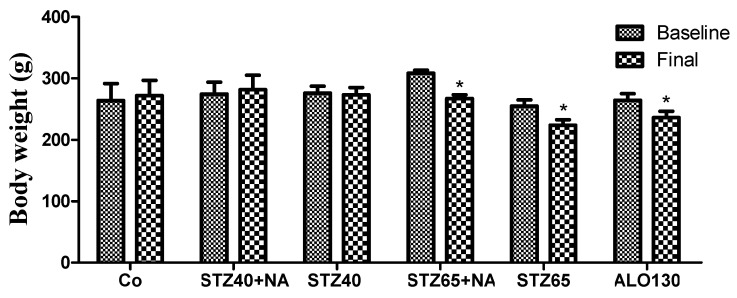
Body weight evolution in female Wistar rats from baseline to pre-sacrifice in the experimental groups (*n* = 7/group). * *p* ≤ 0.05.

Body weight was evaluated at baseline and at the end of the experimental period across all groups Data are expressed as mean ± SD. Statistical analysis was performed using two-way ANOVA with time and treatment as factors, followed by post hoc multiple-comparison testing (Figure 3).

Two-way ANOVA revealed a significant effect of time (F(1,68) = 7.2, *p* = 0.0091), indicating an overall change in body weight during the study period. A significant effect of treatment group was also observed (F(5,68) = 9.5, *p* < 0.0001), demonstrating differences among experimental groups. Importantly, the interaction between time and treatment was statistically significant (F(5,68) = 2.7, *p* = 0.0271), suggesting that weight evolution differed depending on the experimental model.

At baseline (day 0), body weight values were generally comparable across experimental groups. CO group displayed a slight increase in body weight over the experimental period. Similarly, rats receiving the low-dose STZ40+NA protocol maintained body condition and showed a modest weight gain, suggesting good tolerability of this induction method. Rats in STZ40 group experimented neither gain, nor lost weight.

In contrast, animals exposed to more aggressive diabetogenic protocols exhibited weight loss. The alloxan 130 mg/kg group showed the most pronounced reduction, reflecting a severe systemic impact. Weight loss was also evident in STZ65 groups, indifferent to NA protection.

Overall, body weight changes supported the distinction between moderate protocols (STZ–NA, low dose) and severe protocols (alloxan and high-dose STZ±NA), with the latter showing reduced overall tolerance.

### 3.4. Serum Oxidative Stress Markers Evaluation

**Figure 4:**
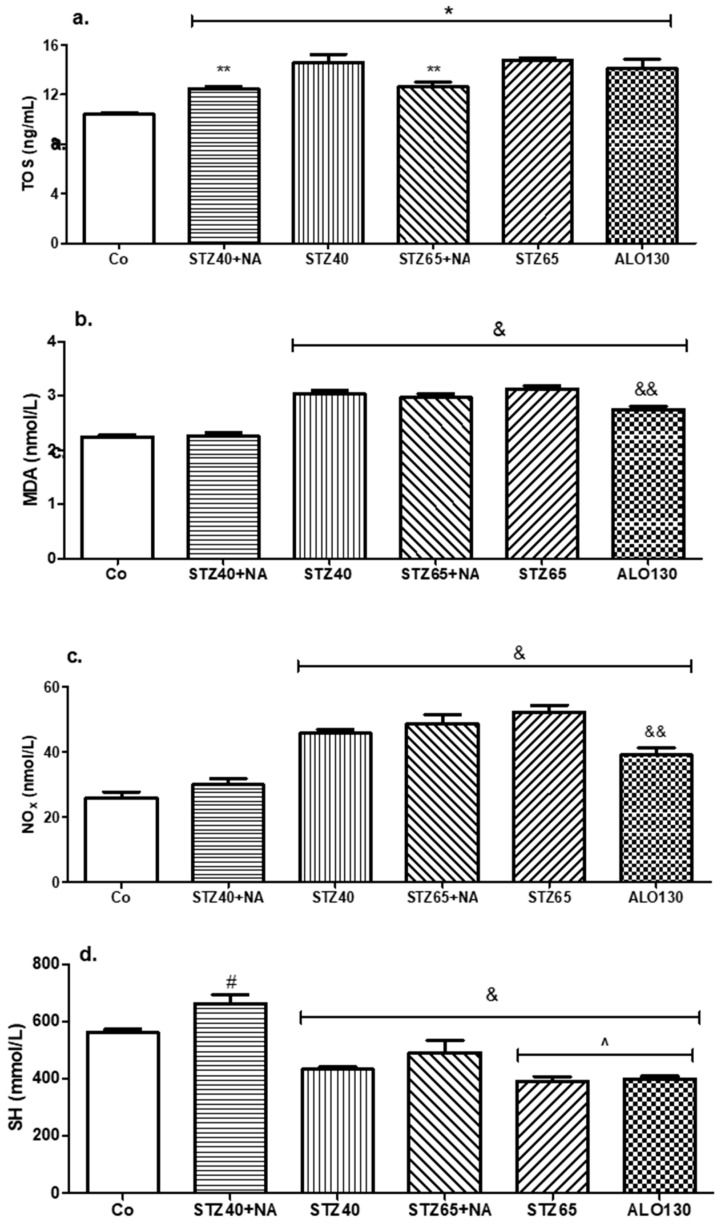
Comparative assessment of oxidative stress markers in experimental diabetes models: (**a**) total oxidative status (TOS): * *p* ≤ 0.05 (Co vs. all treatment groups), ** *p* ≤ 0.05 (STZ40+NA and STZ65+NA significantly lower levels compared to STZ40, STZ65, ALO130); (**b**) malondialdehyde (MDA): ^&^ *p* ≤ 0.05 (Co and STZ40+NA vs. STZ40, STZ65+NA, STZ65, ALO130), ^&&^ *p* ≤ 0.05 (ALO130 vs. STZ40, STZ65+NA, STZ65); (**c**) nitric oxide (NO): ^&^ *p* ≤ 0.05 (Co and STZ40+NA vs. STZ40, STZ65+NA, STZ65, ALO130), ^&&^ *p* ≤ 0.05 (ALO130 vs. STZ40, STZ65+NA, STZ65); (**d**) total thiol groups (SH): ^&^ *p* ≤ 0.05 (Co and STZ40+NA vs. STZ40, STZ65+NA, STZ65, ALO130), ^#^ *p* ≤ 0.05 (STZ40+NA vs. all treatment groups and Co), ^ *p* ≤ 0.05 (STZ65 and ALO130 significantly lower levels vs. STZ40+NA, STZ40, STZ65+NA and Co).

Oxidative stress parameters were assessed by measuring Total Oxidative Status (TOS), Malondialdehyde (MDA), Total Thiol Groups (SH), and Nitric Oxide (NO) in plasma samples. Data are expressed as mean ± SD. Statistical analysis was performed using one-way ANOVA followed by Tukey’s post hoc test, with statistical significance set at *p* < 0.05 (Figure 4).

Total oxidative status showed a significant overall difference among experimental groups (*p* < 0.05). Mean TOS values (μM H_2_O_2_ equivalents/L, mean ± SD) were 11.15 ± 1.36 in the Control group, 12.49 ± 0.51 in STZ40+NA, 14.11 ± 1.45 in STZ40, 13.92 ± 2.25 in STZ65+NA, 13.65 ± 1.27 in STZ65, and 13.29 ± 0.66 in the ALO130 group. All diabetic groups exhibited higher mean TOS values compared with CO.

Malondialdehyde concentrations differed significantly among groups (*p* < 0.0001). Mean MDA levels (nM/mL) were 2.24 ± 0.13 in CO, 2.27 ± 0.12 in STZ40+NA, 2.75 ± 0.11 in STZ40, 3.03 ± 0.14 in STZ65+NA, 2.98 ± 0.24 in STZ65, and 2.81 ± 0.16 in the ALO130 group. MDA values were significantly elevated in STZ40, STZ65+NA, STZ65, and ALO130 groups compared with CO. The STZ40+NA group maintained significantly lower MDA levels compared with STZ-only groups.

Nitric oxide concentrations also showed significant intergroup variation (*p* < 0.0001). Mean NO levels (μmol/L) were 27.41 ± 3.82 in CO, 29.95 ± 5.15 in STZ40+NA, 39.15 ± 4.33 in STZ40, 49.34 ± 7.19 in STZ65+NA, 44.30 ± 5.62 in STZ65, and 41.14 ± 4.44 in the ALO130 group. NO concentrations were significantly increased in STZ40, STZ65+NA, STZ65, and ALO130 groups compared with CO, whereas the STZ40+NA group exhibited attenuated values.

Total thiol concentrations differed significantly among groups (*p* < 0.001). Mean SH levels (μmol/L) were 560.2 ± 26.6 in CO, 661.3 ± 84.1 in STZ40+NA, 397.5 ± 20.8 in STZ40, 514 ± 67 in STZ65+NA, 421.0 ± 35.7 in STZ65, and 396 ± 19 in the ALO130 group. SH levels were significantly reduced in STZ40, STZ65, and ALO130 groups compared with CO. In contrast, the STZ40+NA group maintained higher thiol concentrations relative to STZ-only protocols.

Overall, oxidative stress markers demonstrated increased lipid peroxidation and nitrosative stress accompanied by depletion of thiol antioxidant reserves in diabetic models, with the magnitude of alteration varying according to dose and induction strategy.

### 3.5. Histopathological Findings

Histological examination of hematoxylin–eosin–stained sections revealed protocol-dependent structural alterations in pancreatic, hepatic and renal tissues across experimental groups.

***Pancreas***

**Figure 5:**
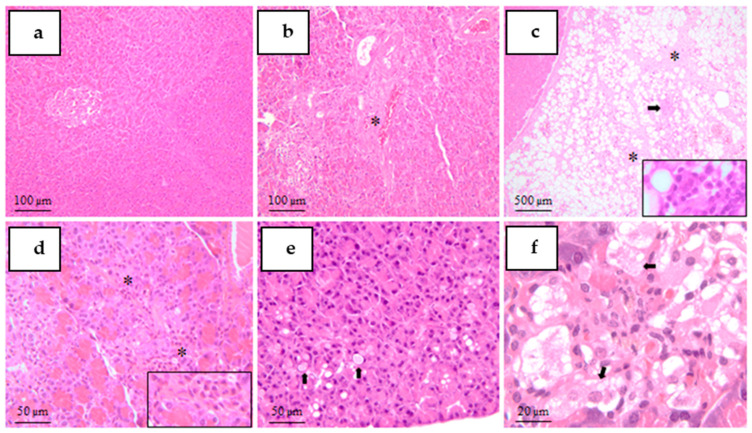
Representative histopathological changes in the pancreas (H&E staining). (**a**)—Normal pancreatic architecture (control group, CO; 10×; scale bar = 100 µm). (**b**)—Pancreatic fibrosis (asterisk) in STZ40 group (10×; scale bar = 100 µm). (**c**)—Peripancreatic adipose tissue with inflammatory cell infiltrate (asterisk) in STZ40+NA group (4×; scale bar = 500 µm). (**d**)—Mixed multifocal inflammatory cell infiltrate composed of polymorphonuclear and mononuclear cells (asterisks) in STZ65 group (20×; scale bar = 50 µm). (**e**)—Exocrine pancreas with multifocal hyaline droplets (arrows) in STZ65+NA group (20×; scale bar = 50 µm). (**f**)—Multifocal vacuolar degeneration of pancreatic islets (arrows) in ALO130 group (40×; scale bar = 20 µm).

Control animals exhibited normal pancreatic histoarchitecture, with well-defined islets of Langerhans and preserved exocrine acini, as shown in Figure 5.

Pancreatic lesions were evident in all diabetogenic protocols, with variable severity. Poorly cellularized, hypocellular islets were observed in STZ40+NA, STZ40, STZ65+NA, STZ65, and ALO130 groups. Rare islets were particularly noted in STZ40 animals.

Pancreatic fibrosis was documented in STZ40 rats, while peripancreatic adipose tissue fibrosis accompanied by inflammatory infiltrates was observed in STZ40+NA animals. Mixed inflammatory cell infiltrates containing polymorphonuclear and mononuclear cells were detected in STZ65 specimens. Increased single-cell death (apoptosis) was also identified in STZ65 animals.

Exocrine pancreatic alterations included multifocal hyaline droplets in STZ65+NA rats. Multifocal vacuolar degeneration of pancreatic islets was most prominent in the Alloxan group, affecting the majority of animals in this protocol. Additionally, rare macrophages containing brown granular pigment were observed in STZ65+NA animals.

Overall, pancreatic structural damage was more pronounced in STZ65 and ALO130 groups, whereas STZ40+NA exhibited comparatively attenuated islet alterations.

***Liver***

**Figure 6:**
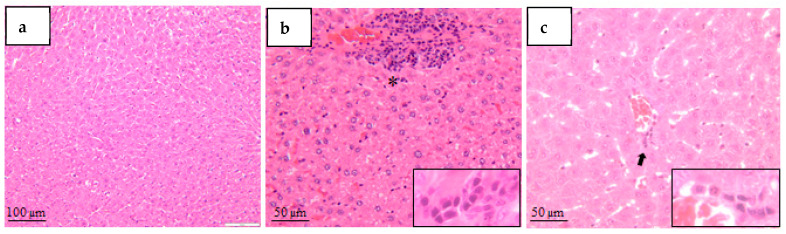
Representative histopathological changes in the liver (H&E staining). (**a**)—Normal hepatic architecture (control group, CO; 10×; scale bar = 100 µm). (**b**)—Focal inflammatory cell infiltrate (asterisk), predominantly mononuclear leukocytes; inset highlights inflammatory infiltrate (STZ+NA, STZ40, STZ65+NA, STZ65, and ALO130 groups; 20×; scale bar = 50 µm). (**c**)—Mixed focal inflammatory infiltrate composed of polymorphonuclear and mononuclear cells (arrow and inset) observed across diabetic groups (20×; scale bar = 50 µm).

Figure 6 shows that control livers displayed preserved lobular architecture with normal hepatocyte morphology and no significant inflammatory or degenerative lesions. Occasional increased mitotic activity was observed in a single control specimen.

Diabetic groups demonstrated predominantly inflammatory and biliary alterations. Minimal focal mononuclear inflammatory infiltrates were identified in STZ+NA, STZ40, STZ65+NA, STZ65, and ALO130 groups, with higher incidence in STZ40+NA and STZ40 animals. Multifocal mild mononuclear infiltrates were detected in STZ65 and ALO130 groups. A polymorphonuclear-dominant inflammatory infiltrate was observed in one Allo specimen.

Biliary hyperplasia was a recurrent finding in diabetic animals. Minimal multifocal biliary hyperplasia was observed in STZ40+NA, STZ40, and STZ65 groups, whereas mild biliary hyperplasia was documented in STZ40, STZ65, and STZ65+NA animals. No extensive necrosis or severe parenchymal destruction was identified.

These findings indicate that hepatic changes were primarily inflammatory and reactive rather than overtly necrotic.

***Kidney***

**Figure 7:**
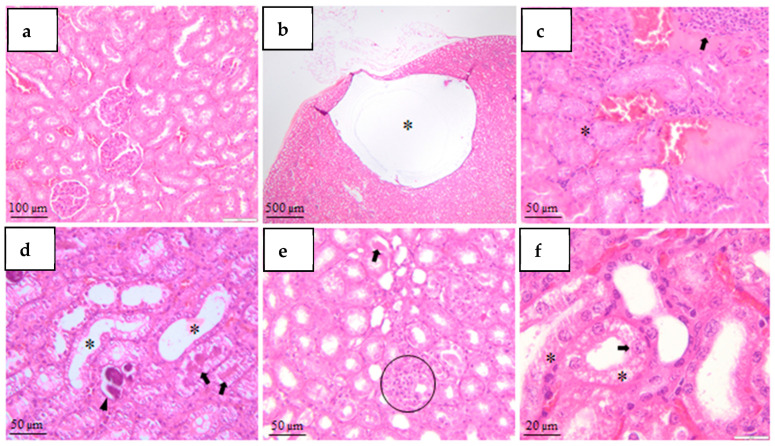
Representative histopathological changes in the kidney (H&E staining). (**a**)—Normal renal architecture with intact glomeruli and preserved tubular epithelium (control group, CO; 10×; scale bar = 100 µm). (**b**)—Renal cyst (asterisk) in STZ40 group (4×; scale bar = 500 µm). (**c**)—Focal progressive nephropathy (asterisk) with inflammatory cell infiltrate composed of mononuclear leukocytes (arrow) in STZ40, STZ65, and ALO130 groups (20×; scale bar = 50 µm). (**d**)—Renal tubular dilation (arrows), granular casts, and medullary mineralization (asterisk) in STZ40 and STZ65 groups (20×; scale bar = 50 µm). (**e**)—Hyaline casts (arrow) and inflammatory cell infiltrate (circled) composed of mononuclear leukocytes in STZ40 group (20×; scale bar = 50 µm). (**f**)—Tubular epithelial cell necrosis (asterisk) and renal tubular epithelial cell vacuolation (arrow) in STZ40 group (40×; scale bar = 20 µm).

Figure 7 shows that control animals exhibited normal renal architecture, characterized by intact glomeruli, preserved Bowman’s capsules, and well-organized tubular epithelium without inflammatory infiltrates or degenerative changes.

In diabetic groups, renal lesions varied in frequency and severity. Minimal focal progressive nephropathy was identified in STZ65, STZ40 and ALO130 groups. Mononuclear inflammatory cell infiltrates were observed in multiple diabetic groups, with higher incidence in STZ 65 and ALO130 animals. Mild mononuclear infiltrates were occasionally detected in STZ40 animals.

Tubular alterations were predominantly associated with STZ-only protocols. Renal tubular epithelial cell vacuolation, hyaline casts, and granular casts were most frequently observed in STZ40 rats. Tubular dilation was noted in STZ40 and STZ65 groups. Minimal tubular epithelial necrosis with evidence of regenerative changes was recorded in STZ40 animals. In addition, glomerular changes characterized by small glomeruli with thickened fibrous Bowman’s capsules were documented in STZ+NA rats. Multifocal medullary mineralization was present in STZ40+NA and STZ40 groups.

Overall, renal histopathological changes were more pronounced in STZ-only groups, while STZ–nicotinamide animals exhibited comparatively milder structural alterations.

Taken together, all induction protocols resulted in distinct diabetic phenotypes. The STZ–NA protocols generated a more moderate and clinically tolerable alteration in glucose metabolism, whereas alloxan and high-dose STZ without protection induced more severe and less stable diabetic states, often associated with pronounced weight loss and reduced suitability for studies requiring stable moderate diabetes models.

## 4. Discussion

The present study provides a structured comparison of streptozotocin (STZ), STZ–nicotinamide (NA), and alloxan-induced diabetes models in female Wistar rats, addressing two relevant gaps in the literature: the underrepresentation of female experimental models and the lack of standardized head-to-head comparisons of commonly used induction protocols.

The focus on female Wistar rats represents an important aspect of the present study. Although male rodents are traditionally preferred in experimental diabetes research to reduce variability associated with the estrous cycle, sex-specific biological differences can meaningfully affect diabetes induction and progression. Available evidence suggests that females may show altered susceptibility to STZ-induced injury, differences in weight evolution, and partially attenuated oxidative and inflammatory responses compared with males, potentially due to estrogen-dependent modulation of insulin sensitivity, beta-cell resilience, mitochondrial function, and redox balance. Accordingly, the inclusion of female animals should be viewed as a strength for translational relevance rather than merely a source of variability [24].

Estrogen-mediated antioxidant effects and modulation of mitochondrial function may alter susceptibility to β-cell injury, suggesting that extrapolation from male-only models may not fully capture sex-specific disease dynamics [25].

Consistent with previous reports, high-dose STZ (65 mg/kg) induced severe and sustained hyperglycemia, reflecting extensive β-cell destruction through DNA alkylation, PARP activation, NAD^+^ depletion, and oxidative stress amplification [26].

Similarly, alloxan (130 mg/kg) produced rapid and pronounced hyperglycemia via redox cycling and acute reactive oxygen species (ROS) generation [27].

However, in agreement with several comparative studies, alloxan-induced diabetes demonstrated a more abrupt metabolic destabilization and greater systemic impact, often associated with weight loss and variability in oxidative markers [28].

The STZ–nicotinamide model generated a milder diabetic phenotype, aligning with prior literature describing its suitability as a type 2 diabetes-like model [29]. Nicotinamide partially protects β-cells by inhibiting excessive PARP activation and preserving intracellular NAD^+^ pools, thereby attenuating ATP depletion and limiting necrosis [30]. This mechanism explains the moderated hyperglycemia and improved metabolic stability observed in our STZ–NA groups. Similar findings have been reported in studies demonstrating that NA co-administration results in partial insulin preservation and reduced oxidative damage compared to STZ alone [15].

The temporal analysis of fasting blood glucose clearly demonstrated that the severity and stability of the diabetic phenotype were strongly dose- and protocol-dependent. High-dose STZ (65 mg/kg) and alloxan (130 mg/kg) produced rapid, sustained, and progressively amplified hyperglycemia, reaching peak divergence at day 7 and remaining markedly elevated at day 14. This pattern is consistent with previously reported data showing that higher STZ doses induce extensive β-cell necrosis through DNA alkylation and PARP overactivation, resulting in near-complete insulin deficiency within the first week post-injection [26].Similarly, alloxan-induced hyperglycemia has been described as abrupt and severe, reflecting acute ROS-mediated β-cell destruction [27].

In contrast, the STZ–nicotinamide groups exhibited moderated glycemic elevation, with values significantly lower than those observed in STZ-only or alloxan groups, particularly in the early phase. This confirms previous reports that nicotinamide partially preserves β-cell function and residual insulin secretion, leading to a more gradual establishment of hyperglycemia [29].

The convergence of STZ65+NA toward STZ40 values by day 14 further supports the modulatory rather than fully protective role of nicotinamide. Collectively, these findings reinforce the concept that STZ+NA protocols more closely resemble a type 2 diabetes-like phenotype characterized by partial β-cell dysfunction rather than complete insulin deficiency.

The oral glucose tolerance test (OGTT) was performed to evaluate dynamic glucose handling and to distinguish between absolute insulin deficiency and impaired glucose tolerance. While fasting glycemia reflects basal metabolic disturbance, OGTT provides insight into the ability of the organism to respond to an acute glycemic challenge, thereby offering functional information regarding residual insulin secretion and peripheral insulin sensitivity.

Although AUC analysis was not performed for the OGTT, the temporal profile clearly demonstrated delayed glucose clearance in the STZ40+NA group, supporting the interpretation of impaired glucose tolerance with residual beta-cell function.

In the present study, control animals demonstrated the expected physiological pattern: a glycemic peak at 30 min followed by progressive normalization. In contrast, the STZ40+NA group exhibited delayed glucose clearance and sustained post-load hyperglycemia, consistent with impaired glucose tolerance rather than complete insulin absence. Similar OGTT alterations have been reported in STZ+NA models and are considered a defining feature of partial β-cell preservation [15].

This supports our intention to demonstrate that the STZ+NA model induces metabolic dysfunction that is moderate and progressive, rather than catastrophic.

Severe STZ-only and alloxan models, by contrast, are typically characterized by minimal post-load recovery due to profound insulin deficiency, a phenomenon widely documented in experimental diabetes research. Therefore, the OGTT served not merely as a confirmatory test of hyperglycemia, but as a mechanistic discriminator between moderate and severe phenotypes.

Body weight analysis further differentiated the induction protocols in terms of systemic metabolic burden. Significant time and treatment interaction confirmed that weight evolution was dependent on the diabetogenic strategy employed. Severe models (STZ65 and alloxan) were associated with marked weight loss, reflecting insulin deficiency–driven catabolism, enhanced lipolysis, proteolysis, and dehydration. Comparable findings have been consistently reported in high-dose STZ and alloxan models, where weight loss is considered an indirect marker of disease severity and metabolic instability [28].

Conversely, animals receiving STZ40+NA maintained body condition and even exhibited modest weight gain, suggesting preserved metabolic adaptability and reduced systemic stress. This observation aligns with previous descriptions of the STZ+NA model as more suitable for long-term studies, pharmacological interventions, and investigations requiring moderate but stable hyperglycemia.

Taken together, the combined evaluation of fasting glycemia, OGTT response, and body weight evolution demonstrates that while all protocols successfully induced diabetes, they differed substantially in severity, stability, and systemic impact. The STZ+nicotinamide model appears to provide a balanced compromise between reproducibility and tolerability, whereas high-dose STZ and alloxan generate aggressive phenotypes characterized by rapid metabolic deterioration and significant catabolic consequences.

Oxidative stress is a central mechanism in the pathogenesis of diabetes mellitus and its complications, acting both as a consequence of hyperglycemia and as a driver of progressive tissue injury. In the present study, all diabetogenic protocols were associated with alterations in oxidative markers, although the magnitude and pattern of change differed depending on dose and induction strategy.

Lipid peroxidation, reflected by increased serum malondialdehyde (MDA) levels, was significantly elevated in STZ-only groups and in the STZ65+NA group compared with control group. These findings are consistent with previous reports demonstrating that STZ induces oxidative damage not only through direct DNA alkylation but also via the generation of ROS secondary to NAD^+^ depletion and mitochondrial dysfunction [13].

Elevated MDA levels have been repeatedly described in STZ-induced diabetic models, correlating with sustained hyperglycemia and enhanced lipid membrane oxidation [31,32].

Interestingly, although alloxan is classically characterized as a potent redox-cycling agent producing superoxide radicals and hydrogen peroxide, MDA levels in our study were not higher than those observed in STZ-only groups [13]. This supports the hypothesis that sustained hyperglycemia-induced oxidative stress may exert a more cumulative lipid-peroxidative effect than the acute ROS burst generated by alloxan administration. Similar observations have been reported in comparative studies where chronic metabolic stress surpassed early oxidative injury in determining long-term lipid peroxidation levels [15].

Nitric oxide (NO) concentrations were also significantly increased in STZ-only and STZ65+NA groups. Hyperglycemia is known to induce inducible nitric oxide synthase (iNOS) expression via Nuclear Factor kappa-light-chain-enhancer of activated B cells (NF-κB) activation, leading to excessive NO production and subsequent peroxynitrite formation [33]. Peroxynitrite contributes to nitrosative stress, protein nitration, and further mitochondrial dysfunction. Elevated NO levels in STZ models have been documented previously and are considered markers of inflammatory activation and endothelial dysfunction in experimental diabetes. The relatively attenuated NO levels observed in the STZ40+NA group may reflect the ability of nicotinamide to inhibit PARP activation and preserve intracellular NAD^+^ pools, thereby limiting NF-κB activation and subsequent iNOS expression. By reducing ATP depletion and oxidative amplification, nicotinamide may attenuate downstream inflammatory and nitrosative signaling pathways [31,34].

Total thiol (SH) concentrations, reflecting non-enzymatic antioxidant reserves—particularly reduced glutathione—were significantly decreased in STZ-only and alloxan groups. Thiol depletion is a well-established indicator of oxidative imbalance in diabetes and reflects increased ROS scavenging and redox buffering consumption [35]. Reduced glutathione depletion has been repeatedly demonstrated in STZ-induced diabetes and correlates with both glycemic severity and tissue injury [31]. The relatively preserved thiol levels in the STZ40+NA group further support the protective role of nicotinamide, which acts as a precursor of NAD^+^ and attenuates PARP overactivation, thereby limiting oxidative amplification [36,37].

The observed pattern—elevated MDA and NO accompanied by reduced SH levels—confirms that oxidative damage and antioxidant depletion occur in parallel and are protocol-dependent. Notably, STZ-only and high-dose protocols produced the most consistent oxidative alterations, whereas STZ–nicotinamide induced a moderated redox imbalance. These findings align with previous comparative studies indicating that STZ–NA models more closely mimic type 2 diabetes-like oxidative profiles characterized by partial β-cell preservation and less abrupt oxidative injury [37].

Taken together, the oxidative stress data reinforce the concept that the choice of induction protocol critically influences not only glycemic severity but also systemic redox status. STZ-only and high-dose strategies generate pronounced lipid peroxidation and nitrosative stress, while nicotinamide co-administration partially attenuates oxidative imbalance. These differences may have important implications for the selection of experimental models in studies targeting antioxidant therapies or inflammatory modulation.

In the present study, chemical induction of diabetes with streptozotocin (STZ) and alloxan (ALO) produced distinct histopathological alterations in liver, kidney, and pancreatic tissues that varied according to dose and co-administration of nicotinamide. These findings align with and extend previous observations that the choice of diabetogenic agent influences not only glycemic severity but also the pattern and severity of tissue remodeling.

The liver of control animals exhibited preserved architecture, consistent with normal physiological morphology. In contrast, all diabetic groups demonstrated mild to moderate inflammatory changes without overt hepatocellular necrosis. Minimal focal mononuclear infiltrates and biliary hyperplasia were recurrent findings, particularly in STZ-treated animals. Such subclinical hepatic alterations have been described in multiple experimental diabetes models. For example, diabetic rats induced with STZ consistently exhibit periportal inflammatory cell infiltrates and reactive bile duct proliferation, even in the absence of significant steatosis or necrosis [31,38,39].

Our observation of a single polymorphonuclear-dominant infiltrate in an alloxan specimen is in line with acute oxidative bursts reported in alloxan models, where superoxide production can transiently recruit polymorphonuclear leukocytes [3].

However, extensive parenchymal destruction was absent, consistent with previous studies showing that early stages of experimental diabetes predominantly involve adaptive and inflammatory responses rather than frank necrosis [38,40].

Renal histopathology revealed dose-dependent tubular and glomerular changes. STZ-only groups exhibited more extensive tubular epithelial vacuolation, cast formation, and minimal necrosis, whereas nicotinamide co-administration was associated with comparatively milder lesions. These findings are consistent with well-established features of early diabetic nephropathy in rodent models, including tubular degenerative changes and interstitial inflammation [26,37].

The appearance of granular and hyaline casts in STZ groups also aligns with proteotoxic tubular stress described in chemically induced diabetes [41].

In contrast, renal changes in STZ–nicotinamide groups were less severe, supporting the protective effect of nicotinamide on β-cell function and secondary metabolic disturbances; similar attenuation of renal injury with nicotinamide has been reported in STZ–NA models, where partial preservation of insulin secretion leads to reduced glomerular hyperfiltration [42].

Pancreatic alterations were expectedly the most prominent, given the direct β-cell toxicity of STZ and alloxan. Hypocellular and poorly defined islets observed across diabetogenic protocols are consistent with β-cell depletion secondary to DNA alkylation (STZ) or ROS-mediated cytotoxicity (ALO) [13,36]. The greater severity of islet damage was observed in STZ65 and alloxan groups.

The presence of mixed polymorphonuclear and mononuclear infiltrates in STZ65 rats suggests ongoing inflammatory activity.

Multifocal vacuolar degeneration of pancreatic islets was particularly prominent in the alloxan group, which is consistent with the rapid oxidative injury mechanism attributed to this compound. Alloxan’s redox cycling generates superoxide radicals and hydroxyl species, leading to abrupt β-cell membrane damage and intracellular edema [13].

The comparatively attenuated pancreatic lesions observed in the STZ40+NA group support the protective role of nicotinamide. Nicotinamide acts as a PARP inhibitor and NAD^+^ precursor, limiting ATP depletion and reducing β-cell necrosis [36,43]. This mechanism explains the preservation of partial islet structure and reduced inflammatory remodeling in the STZ–NA protocol.

Taken together, the histopathological findings confirm that organ injury severity correlates with the intensity of β-cell destruction and systemic metabolic imbalance. STZ-only and high-dose protocols produced more pronounced renal and pancreatic lesions, while STZ–nicotinamide resulted in milder structural changes. Hepatic involvement remained predominantly inflammatory and reactive across all models, suggesting secondary metabolic rather than direct cytotoxic injury.

These results align with previous literature indicating that the choice of diabetogenic protocol not only determines glycemic severity but also influences downstream organ remodeling patterns, which is critical when selecting experimental models for studies targeting microvascular complications or anti-inflammatory therapies.

The present study has several limitations. The experimental duration was limited to 14 days, which reflects early-stage diabetic alterations but does not allow evaluation of long-term microvascular complications. Insulin concentrations and molecular inflammatory markers were not assessed, which may limit mechanistic interpretation. In addition, estrous cycle phase was not controlled and may represent a source of biological variability in female animals. In addition, histopathological assessment was semi-quantitative and did not include digital morphometric analysis of islet number, islet area, or lesion surface, which limits the precision of between-group structural comparisons.

Future research should expand upon these observations by incorporating molecular and mechanistic analyses, including antioxidant enzyme activity, inflammatory mediators, and mitochondrial function, in order to better define protocol-specific redox and inflammatory signatures. Long-term studies are also needed to determine whether early structural and oxidative alterations progress toward clinically relevant diabetic complications. Moreover, given the underrepresentation of female models in experimental diabetology, further investigation into sex-specific metabolic and inflammatory responses may enhance translational applicability.

By providing a structured head-to-head comparison under standardized conditions, the present study contributes to optimizing experimental design and improving reproducibility and translational relevance in diabetes research.

## 5. Conclusions

This comparative study demonstrates that the choice of diabetogenic protocol significantly influences not only glycemic severity but also metabolic stability, oxidative stress burden, and the extent of histopathological remodeling in target organs. High-dose STZ and alloxan models induced sustained hyperglycemia accompanied by pronounced oxidative imbalance and more evident renal and pancreatic structural damage, supporting their suitability for investigations focused on severe insulin deficiency and advanced tissue injury.

In contrast, STZ–nicotinamide protocols generated a moderated diabetic phenotype characterized by partial β-cell preservation, attenuated oxidative stress parameters, improved body weight stability, and comparatively milder organ alterations. The OGTT results further confirmed impaired glucose tolerance rather than complete insulin deficiency in these groups, reinforcing their relevance as type 2 diabetes-like models.

## Data Availability

The data presented in this study are available in this manuscript.
